# Vermiculite Filler Modified with Casein, Chitosan, and Potato Protein as a Flame Retardant for Polyurethane Foams

**DOI:** 10.3390/ijms221910825

**Published:** 2021-10-06

**Authors:** Karolina Miedzińska, Sylwia Członka, Anna Strąkowska, Krzysztof Strzelec

**Affiliations:** Faculty of Chemistry, Institute of Polymer & Dye Technology, Lodz University of Technology, 90-537 Lodz, Poland; sylwia.czlonka@dokt.p.lodz.pl (S.C.); anna.strakowska@p.lodz.pl (A.S.); krzysztof.strzelec@p.lodz.pl (K.S.)

**Keywords:** polyurethane foams, vermiculite, flame retardants, burning behavior, high-ball milling process

## Abstract

In this study, polyurethane (PU) composite foams were modified with 2 wt.% of vermiculite fillers, which were themselves modified with casein, chitosan, and potato protein. The impact of the fillers on selected properties of the obtained composites, including their rheological (foaming behavior, dynamic viscosity), thermal (temperature of thermal decomposition stages), flame-retardant (e.g., limiting oxygen index, ignition time, heat peak release), and mechanical properties (toughness, compressive strength (parallel and perpendicular), flexural strength) were investigated. Among all the modified polyurethane composites, the greatest improvement was noticed in the PU foams filled with vermiculite modified with casein and chitosan. For example, after the addition of modified vermiculite fillers, the foams’ compressive strength was enhanced by ~6–18%, their flexural strength by ~2–10%, and their toughness by ~1–5%. Most importantly, the polyurethane composites filled with vermiculite filler and modified vermiculite fillers exhibited improved flame resistance characteristics (the value of total smoke release was reduced by ~34%, the value of peak heat release was reduced by ~25%).

## 1. Introduction

Polyurethanes (PU) are one of the most widely used groups of polymer materials. They can be produced in various forms, including elastomers, adhesives, coatings, and porous materials, which are divided into two main groups—rigid polyurethane foams (RPUFs) and flexible polyurethane foams (FPUFs) [1,2,3,4]. This enables the use of polyurethane products in many applications, such as thermal and electrical insulation materials, construction, packaging, furniture, or biomedical applications [5,6,7,8,9]. All this is possible thanks to the various raw materials used in the synthesis of polyurethanes [10]. The main raw materials involved in polyurethane chemistry are polyols (compounds containing at least two hydroxyl groups) and polyisocyanates (compounds containing at least two isocyanate groups), and their characteristic feature is the presence of urethane bonds in the main chain of the polymer [11]. During the production of PU materials, additives are also used to ensure the desired material properties, such as blowing agents, surfactants, chain extenders, catalysts, flame retardants, and fillers [11,12]. Rigid polyurethane foams are used as common thermal insulation materials. They are characterized by low thermal conductivity, low apparent density (10–70 kg m^−3^), low brittleness, and high compressive strength. The cellular structure of rigid polyurethane foams has a high closed-cell content. This determines the good dimensional stability of the obtained products and their low thermal conductivity [13]. As for their applications, polyurethane foams are widely used as thermal and sound insulation materials and as low-cost materials in construction, as well as in many industries, including electronics, furniture, refrigeration, and automobiles [10,14,15].

In recent years, the increasing use of polymer materials, their production, and their associated waste resulted in increased environmental requirements. Moreover, the need to reduce the amount of polymer waste and the Sustainable Development Principles have influenced research on the creation of modern modifiers obtained from renewable, natural sources [16,17,18]. Raw materials for the production of polyurethanes, i.e., polyols, and polyisocyanates, are components of mainly petrochemical origin. Current research on rigid polyurethane foams is aimed at replacing petrochemical components with bio-based, natural ones [19]. Currently, some of the most frequently used bioadditives in the synthesis of polyurethane foams are plant-based biopolyols and biofillers [20,21,22,23,24,25].

Studies have shown that biopolyols influence the properties of rigid polyurethane foams. Kurańska et al. [26] used a rapeseed-oil-based biopolyol and observed that the reactivity of polyol systems decreased when increasing the content of biopolyol. Członka et al. [27] used a biopolyol derived from walnut shells and observed improved mechanical properties (compressive strength, flexural strength, and impact strength) in the modified foams. The influence of various fillers on the properties of PU foams have also been described in other studies, such as walnut shells [28], eggshells [22], bamboo stalks [29], hazelnuts [30], and many more.

The flammability of polyurethane foams is very important due to their use in such industries as automobiles, furniture, or construction. Polyurethane foams feature highly porous surfaces, which facilitate the access of oxygen to the materials, and as a result, enhance the combustion process [19]. Therefore, during production, fire retardants are used for these materials. Given the recent addition of new environmental requirements, non-halogen flame retardants are becoming more widely used. These include phosphonates, oxides, phosphates [31], as well as expandable graphite [32,33], inorganic salts [34], and others.

One of the fillers that is also used as a flame retardant is vermiculite, described by the chemical formula (Mg^2+^, Fe^2+^, Fe^3+^)_3_((SiAl)_4_O_10_)OH_2_ 4H_2_O. Structurally, vermiculite is a hydrated mica of variable chemical composition of SiO_2_ (33–39%), MgO (6–30%), Al_2_O_3_ (12–18%), and Fe_2_O_3_ (6–19%). Its advantages include non-toxicity, non-combustibility, and chemical inertia [35]. Similarly to montmorillonite, vermiculite is a 2:1 phyllosilicate, in which the negatively charged aluminosilicate layer is composed of one octahedral sheet sandwiched between two tetrahedral sheets [36,37]. An interesting property of vermiculite is its exfoliation at higher temperatures, due to the loss of water in its interlayers [38]. Vermiculite is known for its flame-retardant properties [39] and that is why can be applied in various polymers, including polypropylene [40,41] or polyurethanes [36,42].

Casein is a bio-based and halogen-free flame retardant, which is a group of related phosphoproteins, described by the chemical formula C_81_H_125_N_22_O_39_P. The advantage that allows it to be used as a flame retardant is its high content of nitrogen and phosphorus [43,44,45,46]. Chitosan is a natural polymer, classified as an amino polysaccharide, composed of a random distribution of β-(1-4)-linked D-glucosamine and N-acetyl-D-glucosamine, with a chemical formula (C_6_H_11_O_4_N)_n_. It is biocompatible and biodegradable. These features increase the interest in chitosan, especially in its potential use in biomedical applications [47,48]. Chitosan contains multi-hydroxyl groups in its structure, which favors the formation of char during the combustion process. This char reduces heat transfer to the polymer and acts as a heat insulator [49,50,51]. Therefore, in this study, it was used as a natural flame retardant. Many proteins exhibit flame-retardant properties, which is why in this study the potato protein was used as a natural flame retardant. It is known that it was used previously as reinforcement filler for polyurethane foams by Członka et al. [52].

There are no known modifications of vermiculite with modifiers such as casein, chitosan, or potato protein, nor of their further use in the synthesis of polyurethane composites. Therefore, this study investigated the influence of the modification of vermiculite as a filler in rigid polyurethane foams on the morphological, mechanical, thermal, and flammability properties of vermiculite.

## 2. Results and Discussion

### 2.1. Fillers Characterization

**Figure 1:**
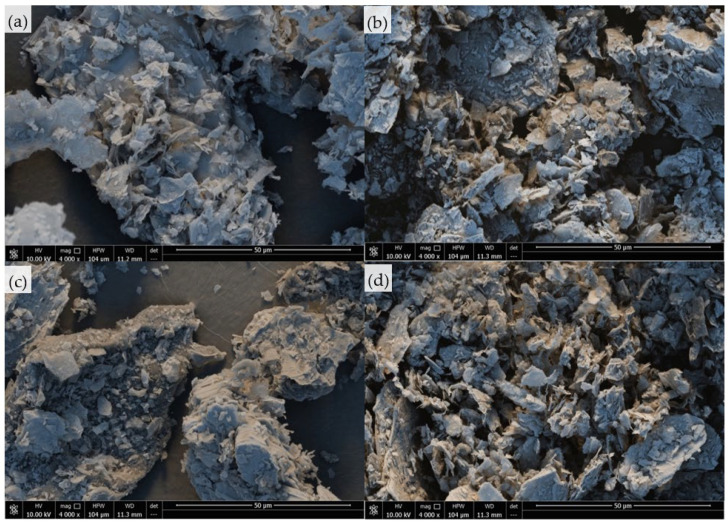
External morphology of (**a**) unmodified vermiculite filler, and vermiculite fillers modified with (**b**) casein, (**c**) chitosan, and (**d**) potato protein.

The external structure of the vermiculite fillers was analyzed using scanning electron microscopy (SEM). The obtained images are presented in Figure 1. It was observed that, before modification, the surface of the vermiculite filler was quite smooth; and after modification, the overall structure of the fillers became rougher and smaller particles were observed.

**Figure 2:**
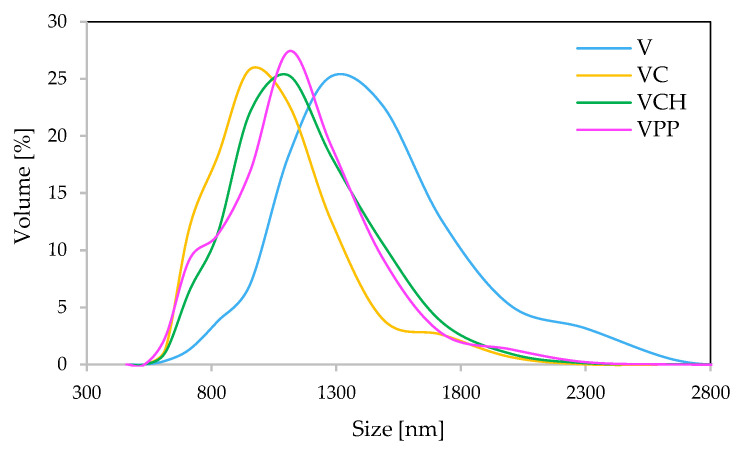
The results of the particle size distribution.

The size of the vermiculite fillers’ particles was assessed by polyol dispersion (0.04 g L^−1^), using the dynamic light scattering method. The particle size distribution of the unmodified vermiculite filler (V) and the vermiculite fillers modified with casein (VC), chitosan (VCH), and potato protein (VPP) are presented in Figure 2. The results indicate that the size of the unmodified and modified vermiculite fillers ranged from 600 to 2600 nm. The highest percentage of particles was observed at ~960 nm for the vermiculite filler modified with casein, ~1100 nm for the vermiculite fillers modified with chitosan and potato protein, and ~1280 nm for the unmodified vermiculite filler.

### 2.2. PU Composites Characterization

**Figure 3:**
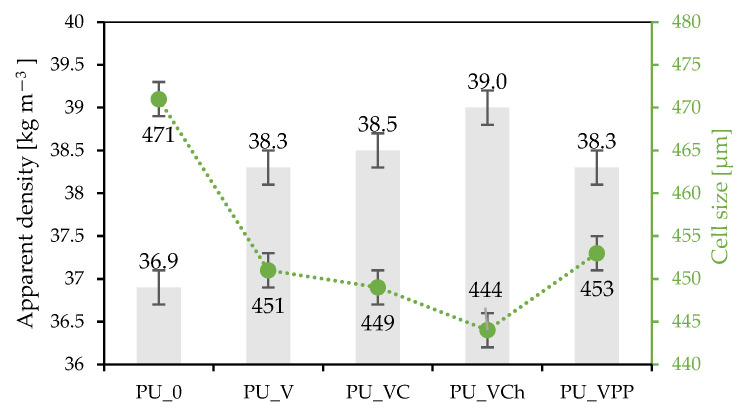
Results of apparent density and average cell size of the PU composites.

As presented in Table 1, the incorporation of vermiculite fillers affected the dynamic viscosity. The increased dynamic viscosity slowed down the free growth of foam cells, which can be observed in the extended processing times, and as a result, also led to an increase in the apparent density of the synthesized composites. When compared with PU_0 (foam without filler addition), after the addition of vermiculite fillers, the dynamic viscosity increased by 8, 9, 14, and 19% respectively, for PU_VPP, PU_V, PU_VC, and PU_VCH. In terms of the characteristic processing times, the addition of vermiculite fillers caused an increase in creaming times of 12, 21, 26, and 33% and an increase in expansion times of 7, 12, 19, and 25% for PU_VPP, PU_V, PU_VC, and PU_VCH, respectively, when compared with PU_0. As presented in Table 1 and Figure 3, the apparent density values increased from 36.9 kg m^−3^ for PU_0 to 38.3 kg m^−3^ for PU_V and PU_VPP, 38.5 kg m^−3^ for PU_VC, and 39.0 kg m^−3^ for PU_VCH. The increase in the viscosity of the polyol mixtures resulted in a slowed expansion of the cells; therefore, the modified foams demonstrated slightly lower average cell sizes. When compared with the reference foam, PU_0, the average cell size decreased from 471 µm to 453 µm for PU_VPP, 451 µm for PU_V, 449 µm for PU_VC, and 444 µm for PU_VCH. However, the differences between the cell sizes of these foams were slight and were often within the error limit.

Figure 3 shows how, with the increase of the apparent density of the synthesized composites, the cells formed in the structure became characterized by smaller sizes. These features would later improve the mechanical properties of the modified polyurethane foams.

When analyzing the data from Table 1, it was observed that for all mentioned parameters (dynamic viscosity, cream and expansion times, and apparent density), the highest values were obtained for the PU_VCH foam.

#### 2.2.1. Morphology of PU Composites

**Figure 4:**
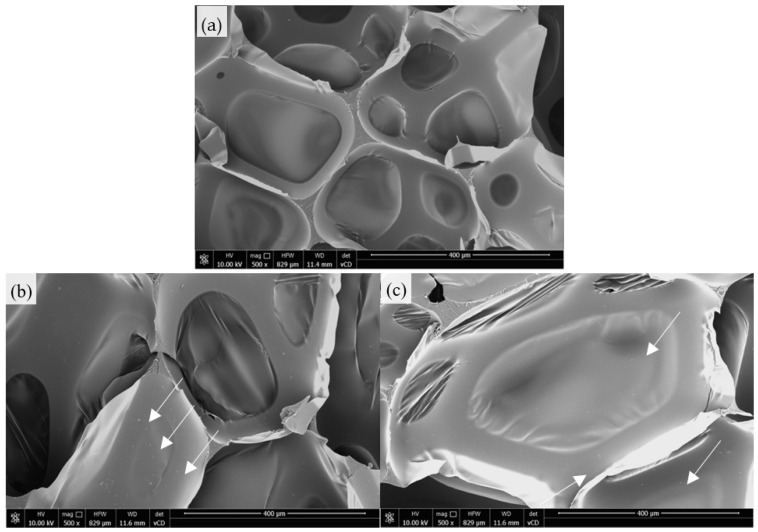

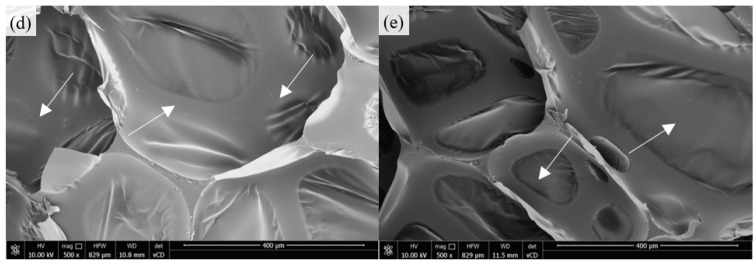
SEM images of (**a**) PU_0, (**b**) PU_V, (**c**) PU_VC, (**d**) PU_VCH, (**e**) PU_VPP.

Figure 4 presents the SEM images of the polyurethane composite foams with different vermiculite fillers. All these foams exhibited well-developed, hexagonal cell structures. In general, the addition of the vermiculite and modified vermiculite fillers improved the morphology of the polyurethane composites—the overall cell structure was uniform, with a great number of regular closed cells. Filler particles can appear in a foam structure two ways: they can be embedded in the cell walls, thus strengthening the structure; or they can appear inside the cells, which is unfavorable as it can lead to the deterioration of the PU structure through the friction between the foam ribs, causing the subsequent deterioration of the structure’s mechanical properties.

During the analysis, in the case of all the analyzed foams, most of the filler particles turned out to be embedded in the cell structure, which resulted in the improvement of the structure’s subsequent mechanical properties. Naturally, there were also filler particles inside the pores, but they constituted a definite minority. In the SEM images presented in Figure 4, the places of occurrence of the filler particles are highlighted by arrows.

#### 2.2.2. Dimensional Stability

The dimensional stability under conditions of lowered and raised temperatures was determined based on the linear changes in the dimensions, length (Δl), width (Δw), and thickness (Δt), of the synthesized polyurethane composites. The conditioning was carried out at −20 °C and +70 °C for 14 days. The results of the completed analysis are presented in Table 2.

The dimensional stability of the polyurethane composite foams indicates that the addition of the vermiculite fillers resulted in negligible changes to the dimensional stability of the modified foams in relation to the reference foam, PU_0. The modified foams demonstrated a generally lower increase in size compared to the PU_0 foam; nevertheless, the difference was insignificant. Following the industrial standard, the polyurethane panels tested at +70 °C were expected to display a linear change of less than 3% and this condition was met by all tested foams [53,54].

#### 2.2.3. Thermogravimetric Analysis (TGA) of Reinforced Polyurethane Composites

**Figure 5:**
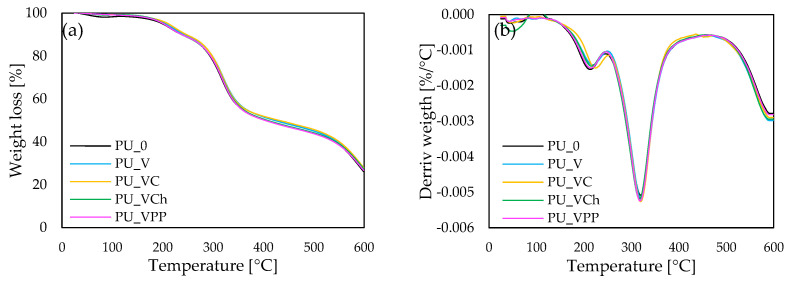
(**a**) Thermogravimetric (TGA) and (**b**) derivative thermogravimetry (DTG) results.

To assess the effect of the vermiculite fillers modified with casein/chitosan/potato protein on the thermal stability of the polyurethane composites, thermogravimetric analysis (TGA) and derivative thermogravimetry analysis (DTG) were performed. During the study, the stages of thermal decomposition were determined. After the measurement, the char residues at the temperature of 600 °C were assessed. The results obtained during the analysis are presented in Figure 5 and summarized in Table 3.

By analyzing the influence of the fillers on the thermal stability of the obtained polyurethane composites, the char residue at the temperature of 600 °C was also measured. When comparing the amount of char residue with the PU_0 result, it can be observed that all the vermiculite fillers (unmodified and modified with casein/chitosan/potato protein) caused an increase in the amount of residue. The content of char residue increased from 26.1% for PU_0 to 26.7, 26.7, 27.5, and 28.0%, respectively, for PU_V, PU_VPP, PU_VCh, and PU_VC. Based on these results, it can be concluded that the application of vermiculite fillers can increase the thermal stability of polyurethane composites.

#### 2.2.4. Mechanical Properties

**Figure 6:**
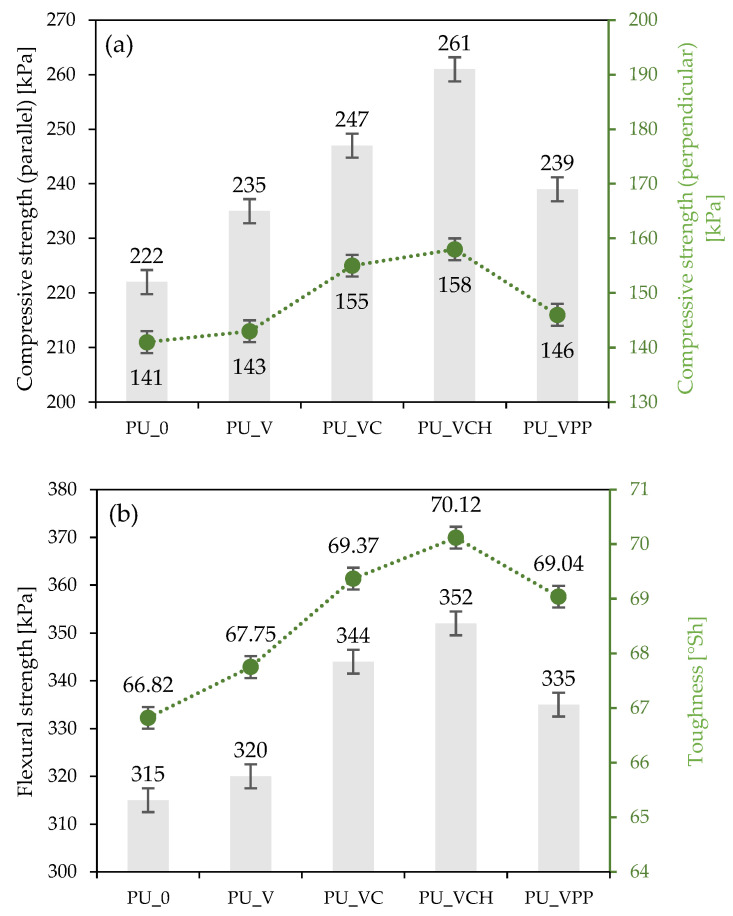
Effect of vermiculite fillers on parallel and perpendicular compressive strength (**a**) and flexural strength and toughness (**b**).

The impact of the vermiculite fillers on the mechanical properties of the synthesized polyurethane composites was determined by measuring the compressive strength at 10% deformation (σ_10%_), flexural strength, and toughness. As presented in Figure 6, the incorporation of vermiculite fillers affected the values of all these parameters. When compared with PU_0, the value of compressive strength (Figure 6a), measured parallel to the direction of foam growth, increased by 6, 8, 11, and 18% for PU_V, PU_VPP, PU_VC, and PU_VCH, respectively. An analogous trend occurred in the case of compressive strength measured perpendicular to the foam growth direction. When compared with PU_0, the value of compressive strength increased by 1, 4, 10, and 12%, respectively for PU_V, PU_VPP, PU_VC, and PU_VCH. Based on these data, it can be observed that vermiculite fillers increased the compressive strength of the obtained polyurethane foams. To avoid the apparent density impact in the mechanical properties, the specific compressive strength was calculated as well (expressed as a ratio of the compressive strength and apparent density of PU composites). The specific compressive strength of PU_0 was 6.02 MPa kg^−1^ m^−3^. After the incorporation of the vermiculite fillers, the value of this parameter increased to 6.14 MPa kg^−1^ m^−3^ for PU_V, 6.24 kg^−1^ m^−3^ for PU_VPP, 6.42 kg^−1^ m^−3^ for PU_VC, and even 6.69 kg^−1^ m^−3^ for PU_VCH.

Figure 6b presents the results of the flexural strength and toughness analysis. In the case of flexural strength, the value of this parameter increased from 315 kPa for PU_0 to 320 kPa for PU_V, 335 kPa for PU_VPP, 344 kPa for PU_VC, and even 352 kPa for PU_VCH. Therefore, the flexural strength of the best foam (PU_VCH) was about 10% better than that of the reference foam. In the toughness analysis, an analogous strengthening of the synthesized composites was observed. When compared with PU_0, the value of toughness increased from 66.82 °Sh to 67.75, 69.04, 69.37, and 70.12 °Sh for PU_V, PU_VPP, PU_VC, and PU_VCH, respectively. Based on the results of the analyses of the mechanical properties, it can be concluded that the application of both the unmodified and the modified vermiculite fillers contributed to the improvement of the mechanical properties. The best results for all the analyzed parameters were recorded for the PU_VCH foam.

#### 2.2.5. Burning Behavior

The flame-retardant properties of the polyurethane composites were performed using a cone calorimeter. The ignition time (IT), peak heat release rate (pHRR), total smoke release (TSR), total heat release (THR), the average yield of CO and CO_2_ (COY and CO_2_Y), and limiting oxygen index (LOI) are presented in Table 4.

**Figure 7:**
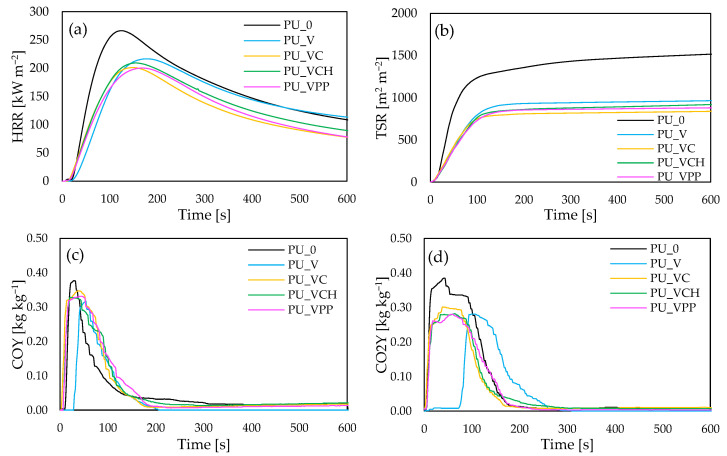
The results of (**a**) peak heat release (pHRR), (**b**) total smoke release (TSR), and an average yield of (**c**) CO and (**d**) CO_2_.

Comparing the foams modified with the vermiculite fillers to the reference one PU_0, it can be observed that the modifications slightly influenced the ignition time (IT). Comparing the ignition time with the reference foam, increases from 4 s for PU_0 to 5 s for PU_V and PU_VCH, and 6 s for PU_VC and PU_VPP, were observed. The flame intensity, related to the low-molecular-weight compound (amines, isocyanates, or olefins) release, was measured by the peak rate of heat release (pHRR). As presented in Figure 7, all the analyzed samples demonstrated one peak of this indicator, and the values of each decreased from 266 kW m^−2^ for PU_0 to 216, 209, 201, and 200 kW m^−2^ for PU_V, PU_VCH, PU_VC, and PU_VPP, respectively. Among all the modified series of polyurethane composites, the lowest pHRR parameter value was observed for PU_VPP, which was about 25% lower than for PU_0. As presented in Figure 7b, the incorporation of vermiculite fillers also resulted in a lower value of total smoke release (TSR). The values of this parameter decreased compared to those of the reference foam from 1515 m m^−1^ to 1150, 1095, 1050, and 1000 m m^−1^ for PU_V, PU_VCH, PU_VPP, and PU_VC, respectively. When analyzing the influence of vermiculite fillers on the total heat release (THR), an analogous relationship can be observed. The values of the THR parameter decreased from 21.7 MJ m^−2^ for the reference foam to 21.1 MJ m^−2^ for PU_V, 20.7 MJ m^−2^ for PU_VCH, 20.1 MJ m^−2^ for PU_VPP, and 19.9 MJ m^−2^ for PU_VC.

As presented in Table 4 and Figure 7c,d the incorporation of vermiculite fillers also reduced the average yield of CO and CO_2_. Compared with the reference foam, PU_0, the average yield of CO and CO_2_ decreased from 0.376 and 0.385 kg kg^−1^ for the reference foam to 0.347 and 0.301 kg kg^−1^ for PU_VC, 0.331 and 0.280 kg kg^−1^ for PU_VPP, 0.328 and 0.282 kg kg^−1^ for PU_VCH, and 0.317 and 0.281 kg kg^−1^ for PU_V. Regarding the limiting oxygen index (LOI), it can be observed that the values of this parameter increased from 20.1% for PU_0 to 21.1, 21.6, 22.5, and 22.8% for PU_V, PU_VCH, PU_VPP, and PU_VCH, respectively.

#### 2.2.6. Water Absorption

**Figure 8:**
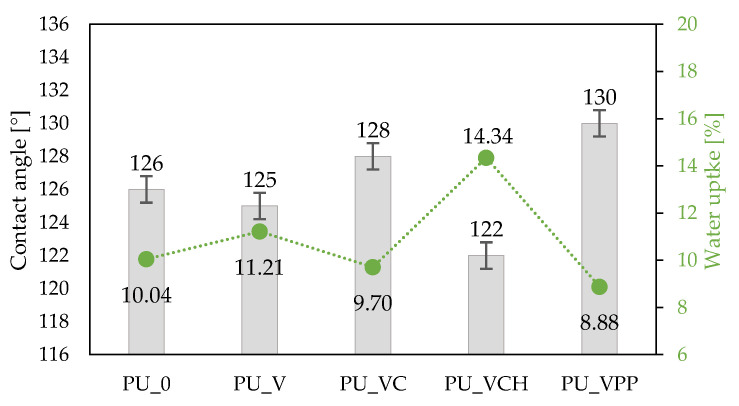
Selected properties of PU foams: contact angle and water uptake results.

**Figure 9:**
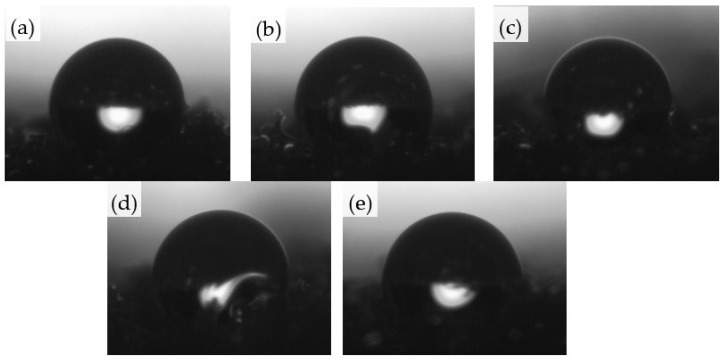
Images of the contact angles measured for (**a**) PU_0, (**b**) PU_V, (**c**) PU-VC, (**d**) PU-VCH, and (**e**) PU_VPP.

The hydrophobic nature of the analyzed foams was assessed using contact angle and water uptake analysis. Water absorption abilities of porous materials depend mainly on their structure (the content of open and closed cells) and their hydrophilic or hydrophobic character. Figure 8 shows the contact angle and water uptake results. When compared with PU_0 (10.04%), the water uptake of the foams increased for PU_V (11.21%) and PU_VCH (14.34%) while the values of the contact angles decreased from 126° for PU_0 to 125° for PU_V, and 122° for PU_VCH. In the case of the other foams, the water absorption decreased (to 9.70% for PU_VC and 8.88% for PU_VPP) while the contact angle values increased (to 128° for PU_VC and 130° for PU_VPP, respectively). This may be related to the fact that unmodified vermiculite and chitosan exhibit hydrophilic properties [55,56]. On the other hand, the remaining modifiers (casein and potato protein) contain many hydrophobic groups that can reduce the water absorption of the obtained composites [57,58]. Figure 9 presents the drops on the surface of the analyzed foams during the examination of the contact angles.

## 3. Materials and Methods

### 3.1. Materials

- Polymeric diphenylmethane diisocyanate with a brand name of Purocyn B, purchased from Purinova Company (Bydgoszcz, Poland);
- Polyether polyol with a brand name of Stepanpol PS-2352, purchased from Stepan Company (Northfield, IL, USA);
- Catalysts: Kosmos 33 (potassium acetate) and Kosmos 75 (potassium octoate), purchased from Evonik Industry (Essen, Germany);
- Surfactant: Tegostab B8513 (silicone-based surfactant), purchased from Evonik Industry (Essen, Germany);
- Blowing agents: pentane and cyclopentane, purchased from Sigma-Aldrich Corporation (Saint Louis, MO, USA);
- Modifiers:
  ○ Vermiculite, purchased from Sigma-Aldrich Corporation (Saint Louis, MO, USA),
  ○ Casein, purchased from Sigma-Aldrich Corporation (Saint Louis, MO, USA),
  ○ Chitosan, purchased from Sigma-Aldrich Corporation (Saint Louis, MO, USA),
  ○ Potato protein, obtained from local company (PEPEES S.A., Łomża, Poland).

### 3.2. Methods and Instruments

The size of the filler particles in polyol dispersion (0.04 g L^−1^) was determined with the dynamic light scattering method after their treatment with ultrasound (1 h), using a Zetasizer NANOS90 instrument (Malvern Instruments Ltd., Malvern, UK). The measurement was evaluated at 3 s intervals. An average of 30 individual scans were obtained and the average spectrum was presented. The measurement was performed three times for each sample.

The viscosity of the polyol systems was assessed using a Viscometer DVII+ (Brookfield, Germany), according to the standard ISO 2555.

The cell size distribution and morphology were analyzed based on the cellular structure images taken using the JEOL JSM-5500 LV scanning electron microscope (JEOL LTD, Akishima, Tokyo, Japan). The microscopic analysis was conducted in a high-vacuum mode and at an accelerating voltage of 10 kV. The samples were scanned in a parallel direction to the foam growth. The pore size distribution and pore diameters were assessed using ImageJ software (Media Cybernetics Inc., Rockville, MD, USA).

The apparent density was measured in accordance with the standard ASTM D1622 (equivalent to ISO 845), as the ratio of sample’s weight to its volume. The density was measured on five samples of each series of foams and expressed as an average.

The dimensional stability of the analyzed polyurethane composites was determined in accordance with the standard ASTM D2126, which is equivalent to ISO 2796. The dimensional stability under conditions of lowered and raised temperatures was determined based on the linear changes in dimensions of the synthesized polyurethane composites. The conditioning was carried out at −20 °C and +70 °C for 14 days.

The three-bonding test was performed according to the standard ASTM D7264 (equivalent to ISO 178) using the Zwick Z100 Testing Machine (Zwick/Roell Group, Ulm, Germany). The samples were bent at a speed of 2 mm min^−1^. For each foam series, at least five measurements were made. The obtained flexural stress at the break results for each sample was expressed as a mean value and averaged.

The compressive strength (σ_10%_) was assessed according to the standard ASTM D1621 (equivalent to ISO 844). The analysis was conducted using the Zwick Z100 Testing Machine (Zwick/Roell Group, Ulm, Germany) with a load cell of 2 kN and a speed of 2 mm min^−1^. The compressive strength was determined as a ratio of the load causing 10% deformation of the cross-section of the analyzed samples, both parallel and perpendicular to the square surface. The compressive strength was measured on at least five samples of each series and expressed as an average.

The toughness of the polyurethane foams was determined using the Shore method, in accordance with the standard ISO 868. During the experiment, the Shore hardness tester (00 types) by Zwick/Roell, equipped with a ball indenter with a diameter of 1.2 mm, was used. During the testing of each series of foams, at least 15 measurements were performed, and the result was expressed as an average.

The thermal stability was analyzed using a Mettler Toledo Thermogravimetric Analyzer TGA/DSC1 (Mettler Toledo, Greifensee, Switzerland). The study included the analysis of the mass change as a function of temperature during the thermal decomposition of the polyurethane foams.

The burning behavior and flame-retardant properties were analyzed using a cone calorimeter following the standard ISO 5660 in S.Z.T.K. TAPS (Maciej Kowalski Company, Saugus, Poland). The measurement was carried out on three samples for each foam series and expressed as an average.

The surface hydrophobicity was determined by contact angle measurements using the sessile drop method. The examination was performed using a manual contact angle goniometer with an OS-45D optical system (Oscar, Taiwan) to capture the shape of liquid on the solid surface. Water drops of 1 µL were deposited using a micrometer syringe fitted with a stainless-steel needle onto the flat surface neatly cut out from the inside of the foam. The contact angles were measured at least ten times on each sample and averaged.

The water absorption of the foams was measured according to the standard ASTM D2842 (equivalent to ISO 2896). The tested samples were dried at 80 °C for 1 h and then weighed. Subsequently, the samples were immersed in distilled water at a depth of 1 cm for 24 h. Next, the samples were removed from the water, held vertically for 10 s, and dried between sheets of dry filter paper at 10 s and weighed again. The water absorption was measured in five samples of each foam and expressed as an average.

### 3.3. Filler Modification

Before incorporation to the polyol system, the ground vermiculite fillers were modified with casein/chitosan/potato protein using a high-energy ball milling process. The vermiculite fillers were mixed with either casein, chitosan, or potato protein powder, respectively (vermiculite filler weight to casein/chitosan/potato protein weight ratio = 1:1) and milled using a high-energy ball milling process with a PULVERISETTE 5 Classic Line planetary ball mill (Fritsch, Idar-Oberstein, Germany) (30 min, 3000 rpm, ball weight to powder weight ratio = 12:1). The vermiculite fillers modified with casein/chitosan/potato protein were used as reinforcing fillers in the synthesis of the polyurethane composites.

### 3.4. Polyurethane Composites Synthesis

**Figure 10:**
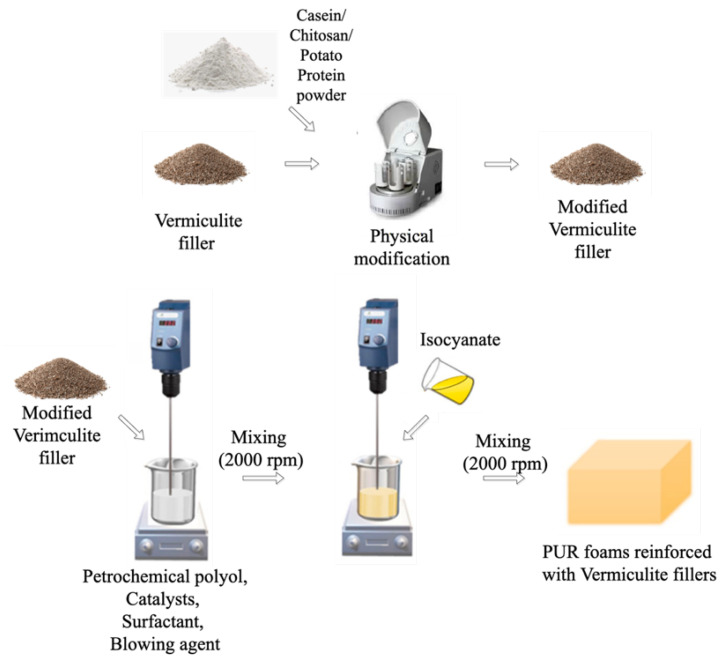
Schematic procedure of the synthesis of PU composites reinforced with vermiculite filler modified with casein/chitosan/potato protein.

The calculated amounts of polyol (Stepanpol PS2352), fillers, catalysts (Kosmos 33, Kosmos 75), surfactant (Tegostab B8513), blowing agent (pentane/cyclopentane), and water were placed in a container and mixed thoroughly (60 s, 2000 rpm). Next, the isocyanate compound was added into the container with thorough stirring (30 s, 2000 rpm). In line with the supplier information, the isocyanate was mixed in a 100:160 ratio (polyol to isocyanate) to ensure a complete reaction between the components. The obtained polyurethane composites were cured for 48 h at room temperature. All the compositions of the prepared composites are presented in Table 5 (where the ‘-’ sign means that the selected filler has not been added). The schematic procedure of the synthesis of polyurethane composites is presented in Figure 10.

## 4. Conclusions

Polyurethane foams were successfully reinforced using an unmodified vermiculite filler and vermiculite fillers modified with casein/chitosan/potato protein. The addition of all these fillers into the polyol systems increased their dynamic viscosity and resulted in the elongation of the characteristic processing times of the PU synthesis. The sizes of the unmodified and modified vermiculite fillers ranged from 600 to 2600 nm. The analysis of the impact of the fillers used indicates that the incorporation of 2 wt.% of vermiculite filler and modified vermiculite fillers affects the cellular structure and apparent density of polyurethane composites and their further mechanical, thermal, and application properties. The SEM images showed that the filler particles were embedded in the PU structure. The best results of the experiments were obtained from the polyurethane foams with the addition of vermiculite fillers modified with casein and chitosan. For example, the incorporation of 2 wt.% of vermiculite filler modified with chitosan provided the polyurethane foams with better compressive strength (parallel—improvement by ~18%, perpendicular—improvement by ~12%), flexural strength (improvement by ~12%), and toughness (improvement by ~5%). The incorporation of 2 wt.% of vermiculite filler modified with casein provided the polyurethane foams with improved flame-retardant properties, such as peak heat rate release (reduction by ~24%), total smoke release (reduction by ~34%), and total heat release (reduction by ~8%). Moreover, these foams may show greater thermal stability and a higher content of char residues. The results presented here confirm that the application of vermiculite and vermiculite modified with casein/chitosan/potato protein can be effectively used as natural fillers in the production of PU products.

In conclusion, the advantages of the modified foams obtained in this study include the improvement of the mechanical and flammability properties of polyurethane foams.

## Figures and Tables

**Table 1:** Rheological properties of polyurethane composites.

	PU_0	PU_V	PU_VC	PU_VCH	PU_VPP
Dynamic viscosity at 10 rpm (mPa·s)	850 ± 8	930 ± 9	970 ± 8	1010 ± 8	920 ± 7
Cream time (s)	39 ± 3	47 ± 5	49 ± 3	52 ± 4	44 ± 4
Expansion time (s)	260 ± 6	290 ± 4	309 ± 8	324 ± 7	278 ± 8
Apparent density (kg m^−3^)	36.9 ± 0.7	38.3 ± 0.5	38.5 ± 0.7	39.0 ± 0.8	38.3 ± 0.6
Average cell size (µm)	471 ± 5	451 ± 3	449 ± 5	444 ± 4	453 ± 5

**Table 2:** Changes in linear dimensions, mass, and volume, after conditioning at −20 °C and +70 °C, of polyurethane composites.

Sample	Temperature of −20 °C			Temperature of +70 °C		
	Δl (%)	Δw (%)	Δt (%)	Δl (%)	Δw (%)	Δt (%)
PU_0	1.80 ± 0.01	1.83 ± 0.01	1.77 ± 0.01	1.93 ± 0.01	1.89 ± 0.01	1. 74 ± 0.01
PU_V	1.76 ± 0.01	1.80 ± 0.01	1.79 ± 0.01	1.79 ± 0.01	1.68 ± 0.01	1.73 ± 0.01
PU_VC	1.69 ± 0.01	1.74 ± 0.01	1.73 ± 0.01	1.74 ± 0.01	1.83 ± 0.01	1.66 ± 0.01
PU_VCH	1.73 ± 0.01	1.68 ± 0.01	1.69 ± 0.01	1.77 ± 0.01	1.70 ± 0.01	1.69 ± 0.01
PU_VPP	1.70 ± 0.01	1.77 ± 0.01	1.64 ± 0.01	1.66 ± 0.01	1.82 ± 0.01	1.70 ± 0.01

**Table 3:** The results of the thermal stability analysis of the polyurethane composites.

Sample	T_max_ (°C)			Char Residue (wt.%) at 600 °C
	1st Stage	2nd Stage	3rd Stage	
PU_0	213 ± 3	319 ± 4	591 ± 5	26.1 ± 0.1
PU_V	219 ± 4			26.7 ± 0.1
PU_VC	225 ± 2			28.0 ± 0.2
PU_VCh	215 ± 3			27.5 ± 0.1
PU_VPP	215 ± 2			26.7 ± 0.2

**Table 4:** Flame-retardant properties of PU composites.

Sample	IT (s)	pHRR (kW m^−2^)	TSR (m^2^ m^−2^)	THR (MJ m^−2^)	COY (kg kg^−1^)	CO_2_Y (kg kg^−1^)	LOI (%)
PU_0	4	266	1515	21.7	0.376	0.385	20.1
PU_V	5	216	1150	21.1	0.317	0.281	21.2
PU_VC	6	201	1000	19.9	0.347	0.301	22.8
PU_VCH	5	209	1095	20.7	0.328	0.282	21.6
PU_VPP	6	200	1050	20.1	0.331	0.280	22.5

**Table 5:** Composition of polyurethane foams.

System	Compound	Content (wt. % to Polyol)				
		PU_0	PU_V	PU_VC	PU_VCh	PU_VPP
Polyol system	Stepanpol PS2352	100				
	Pentane/cyclopentane	11				
	Kosmos 33	5				
	Tegostab B8513	2.5				
	Kosmos 77	0.8				
	Water	0.5				
Filler	Vermiculite	-	2	-	-	-
	Vermiculite functionalized with casein	-	-	2	-	-
	Vermiculite functionalized with chitosan	-	-	-	2	-
	Vermiculite functionalized with potato protein	-	-	-	-	2
Isocyanate system	Purocyn B	160				
